# Body Posture Asymmetry in Prematurely Born Children at Six Years of Age

**DOI:** 10.1155/2017/9302520

**Published:** 2017-10-18

**Authors:** Katarzyna Walicka-Cupryś, Justyna Drzał-Grabiec, Maciej Rachwał, Paweł Piwoński, Lidia Perenc, Łukasz Przygoda, Katarzyna Zajkiewicz

**Affiliations:** ^1^Medical Faculty, Institute of Physiotherapy, University of Rzeszow, Rejtana 16c, 35-959 Rzeszów, Poland; ^2^Centre for Innovative Research in Medical and Natural Sciences, Medical Faculty, University of Rzeszow, Warzywna 1a, 35-310 Rzeszów, Poland

## Abstract

**Aims:**

The purpose of the study was to assess body posture asymmetries in the standing and sitting position in prematurely born children at six years of age.

**Study Design and Subjects:**

We measured trunk symmetry in coronal plane. The study was carried out in a group of 101 children, aged 6-7 years, mean age of 6.63, including 50 preterm children born at gestational age <32 weeks (preterm group) and 51 full-term children (control group).

**Outcome Measures:**

Trunk symmetry in coronal plane was measured using photogrammetric technique with Mora 4G CQ Elektronik. The subjects were examined in standing and sitting position. Statistical analyses were carried out using Shapiro-Wilk *W*-test, Student's *t*-test, Mann–Whitney *U* test, and Pearson's chi-squared test. Statistical significance was assumed at *p* < 0.05.

**Results:**

No significant differences were found between the groups in the asymmetries identified in the relevant anthropometric points, relative to the position assumed during the examination or to the subjects' sex.

**Conclusions:**

There are no significant differences in body posture in the coronal plane, between preterm children and full-term children. Premature birth does not have adverse effects related to body posture asymmetry in preterm children at the age of six.

## 1. Introduction

Due to advances in perinatal and neonatal care, survival rates for preterm infants have increased [1]; however, the risk of neurodevelopmental impairment remains high [2].

Neonatal posture requires a number of active postural control mechanisms, that is, neuromotor functions, which allow controlling its body posture at rest, during displacement and during active movements [3]. Postural control is intimately linked to motor control: dynamic motor actions cannot be performed without first stabilising body posture [4].

The third trimester in the uterus, which is missed in part or in whole by premature infants, promotes the ideal, flexed position when the infant is crowded by the uterine environment and experiences rapid brain growth, mediating flexion (arms and legs bent and trunk tucked forward), and midline orientation [5]. Positioning in physiological flexion (flexion of the shoulders, hips, and knees, scapular protraction, and posterior pelvic tilt) is the ideal position of the newborn, as it promotes proper joint alignment and symmetry, supports neuromuscular development, and promotes self-soothing and behavioural organization [6]. However, premature infants lack tonal responses and strength at birth, and they often assume extended (straight) positioning of the neck, back, and extremities [7]. Extended positioning can affect acquisition of developmental motor skills and hinder self-regulation [8] and may interfere with oral feeding skills. One study found that children born preterm were more likely to demonstrate extension in the trunk which interfered with sitting posture and significantly influenced mobility, promoted asymmetry, and decreased hand function at 1 year of age [9]. Most studies into body asymmetry in preterm children focus on the period of infancy, yet there are no research reports discussing the related effects in the development of body posture in older children.

The aim of our study was designed to assess body posture asymmetries in the standing and sitting position in prematurely born children at six years of age.

## 2. Methods

### 2.1. Participants

The study examined a group of 101 children, aged 6-7 years, mean age of 6.63, including 50 preterm children: 25 boys and 25 girls (preterm group) and 51 full-term children: 22 boys and 29 girls (control group). Children with neurologic or orthopaedic disorders affecting the ability to assume vertical posture without assistance were excluded from the study. The following criteria for including into the study group were adopted: guardians' and children's consent for participation, birth before gestational age of 32 weeks, and lack of neurologic and orthopaedic disorders affecting body posture. Inclusion criteria for the controls were as follows: guardians' and children's consent for participation, lack of neurologic and orthopaedic disorders affecting body posture, age matching that of the study group, and birth at gestational age after 36 and before 42 weeks. The study was conducted in 2016, in south-eastern Poland, at the Centre for Innovative Research in Medical and Natural Sciences operating at the University of Rzeszów.

### 2.2. Anthropometric Measurements

All the measurements were performed on the same day, starting with anthropometric measurements. Body height was measured with Seca 213 mobile stadiometer, with an accuracy of 0.1 cm. Body mass was measured using electronic scale OMRON BF 500, with an accuracy of 0.1 kg. The measurements were performed in standard conditions; children in underwear and barefoot were standing in upright position, without bending knees. Anthropometric measures of both groups are shown in Table 1.

### 2.3. Body Posture Measurements

Body posture was examined using photogrammetry technique based on projection moiré phenomenon, Mora 4G CQ Elektronik [10].

Research has confirmed that results obtained by photogrammetric method are very close to X-ray outcomes [11, 12] and reproducible [13].

Before the measurement, anthropometric points, used later to calculate the relevant parameters, were marked on each subject's body. The anthropometric points were determined by palpation by a physiotherapist with 10 years of experience and extensive practice in photogrammetric measurements. Following the palpation of the points (spinous processes, lower corners of the scapulae, peak of kyphosis, the deepest point of lordosis, transition of kyphosis into lordosis, and posterior iliac spines), they were marked with a dermatograph, the procedure being consistent with other similar studies [14–17]. The image was recorded after marking all the essential points and positioning the subject with his or her back to the camera, in habitual standing posture with straight knees and gaze oriented forward. The computer registered around a dozen images. During the next recording of spinal curvatures the subject was sitting on a stool, with feet against the floor, in an unrestrained position, without spine adjustments, with gaze oriented forward. This assessment was performed because the sitting posture is a preferred position for examining the rib or loin hump during school screening as it demonstrates the best correlation with the spinal deformity exposing the real trunk asymmetry [18]. Then, the image capturing correct positioning of the subject, in the habitual position without twisting of the trunk or pelvis, was selected, and the anthropometric points were transferred onto a photogram on the computer screen. Based on the marked points, the computer defined the parameters describing the body posture by assessing the distance of the selected points from the camera.

The subject is positioned at a distance of 2.6 m from the camera while the device projects lines of strictly defined parameters onto his/her back, allowing a spatial image to be obtained. These lines reach the subject's back at a specific angle and are distorted depending on the distance of a given point from the device. The computer records line image distortions, and numerical algorithms are used to convert these into a contour map of the surface. In optics, the physical basis of this method is called the moiré phenomenon [19].

**Figure 1:**
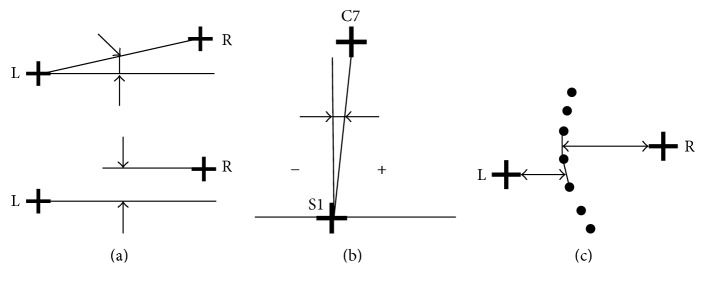
A method of determining the following parameters: (a) differences in the height of shoulders, scapulae and pelvis; (b) coronal inclination of the trunk; (c) difference in distances from the lower corners of the scapulae to the spine.

We analysed the parameters presented in Figure 1:

  CIT [degree]: coronal inclination of the trunk;
  DHS [mm]: difference in the height of shoulders;
  DHCS [mm]: difference in the height of the lower corners of the scapulae;
  DDCS [mm]: difference in the distances from the lower corners of the scapulae to the spine;
  DHP [mm]: difference in the height of the pelvis;
  SA [mm]: maximum deflection of the line connecting the spinous processes from C7-S1 line.

### 2.4. Statistics

Statistical analyses of the collected material were performed using Statistica 10.0 from StatSoft. Both parametric and nonparametric tests were applied in the analysis of the variables. The choice of parametric test depended on the fulfilment of its basic assumptions, that is, conformity of the distributions of the examined variables with normal distribution, which was verified with Shapiro-Wilk *W*-test. Descriptive statistics, calculated for all numerical variables, included the mean, median, and standard deviation. Assessment of differences in the average value of a numerical characteristic in the two populations was performed with Student's *t*-test for independent variables, or alternatively, with nonparametric Mann–Whitney *U* test. Analysis of qualitative data was carried out using Pearson's chi-squared test. Statistical significance was assumed at *p* < 0.05.

### 2.5. Ethics

The study was approved by Bioethics commission at the Faculty of Medicine, University of Rzeszów, Poland. The children's parents gave their written informed consent to their children' participation in the study.

## 3. Results

Calculations were performed to determine coronal plane parameters of trunk posture and their possible asymmetries in preterm and full-term children at the age of 6 years.

Analyses of body posture parameters related to asymmetries in trunk inclination, position of the shoulders, position of the scapulae and their distance from the spine, position of the pelvis, and shape of the spine in the coronal plane have shown no significant differences between the group of preterm children and the group of full-term children, regardless of the assumed posture (Table 2).

The findings show that, in comparison to the controls, the female preterm subjects presented with slightly higher values of parameters related to asymmetry in the inclination of trunk (CIT) and shoulders (DHS), as well as distance between the scapulae and the spine (DDCS), and with lower asymmetries in the height of the scapulae (DHCS) and the pelvis (DHP). The identified differences were statistically insignificant, both in standing and in sitting position (Table 3).

Analysis of data representing trunk posture parameters in boys showed mild differences between the groups related to asymmetry, regardless of the position during measurements. However, no statistically significant differences were identified in the parameters pertaining to body posture symmetry (Table 4).

In view of the lack of asymmetry related differences between the girls and the boys, relative to their birth time, the analyses of asymmetry orientation took into account only gestational age at birth.

The findings show that while standing the subjects in both groups more often present with lower position of the right shoulder, scapula and pelvis, the left scapula closer to the spine, and leftward deflection of spinous processes from C7-S1 line. Similar tendencies are observed in sitting position, and both groups are also found with more frequent rightward inclination of the trunk while the preterm children, in the standing position, are more likely to present with leftward inclination of the trunk (Table 5).

## 4. Discussion

The present study has not identified statistically significant differences, in parameters defining postural symmetry, between preterm children and those born at term. Regardless of the birth time and position during measurement, subjects in the entire group more often present with lower position of the right shoulder, scapula and pelvis, the left scapula closer to the spine, and leftward deflection of spinous processes from C7-S1 line as well as rightward inclination of the trunk.

In the literature there are no reports related to parameters defining body posture in prematurely born children; researchers have previously focused only on assessing postural control or generally body posture in the relevant group. These studies, however, mainly relate to infants.

Findings of previous studies from 1986 report differences in postural control observed in infants. According to authors the number of atypical qualitative posture and mobility characteristics demonstrated by preterm infants was significantly greater than full-term infants ranging in age from 1 day to 9 months but not for those from 10 to 12 months of age. The predominant difference between the two samples was the presence of neck hyperextension and arching of the trunk which occurred significantly more often in the preterm infants [20]. De Vries and De Groot, in a study of dystonia in two-and-a-half-year-old premature infants and correctly born peers, reported lower rotational efficiency of the torso, as well as poorer hand and arm efficiency [21].

However, these differences are less distinctive in older children, closer in age to the group assessed in the present study. According to Kluenter et al., static and dynamic postural control did not significantly differ in full-term and preterm children, with very low birth weight, at 7 years of age [22].

Although, in statistical terms, the preterm infants presented a sequence in the development of postural control similar to that of the full-term infants and were within the age range at each level showed by Pountney et al. for normal infants, they presented a different trend in the acquisition of motor abilities [23]. Pin et al. mention the differences in the movement behaviour of premature babies and peers born correctly. Uneven development of movement skills* can be observed* in different positions. [24].

According to De Groot et al. asymmetries in the motility and posture of preterm infants after term age are a common finding, but their diagnostic and prognostic significance has proved to be difficult to interpret. It has been claimed that if asymmetry is of central origin, then it should be most prominently detectable in infantile reactions that persist beyond the age when they should have disappeared [25]. What is more, Liu et al. point to the existence of structural asymmetry in the network of locomotor neurons and language networks. This asymmetry occurs in healthy preterm neonates at term equivalent age earlier than the development of speech and hand performance [26].

The preterm children had poorer muscle tone [27, 28]. Posture and postural changes are ultimately the result of muscle activity. Postural muscle activation is generated before movement is initiated and involves constant change and accommodation. Adequate postural control and muscle tone are needed [29].

For the clinical study of posture, spontaneous and elicited behaviour which cause active muscle power (AMP) and passive muscle tone (PMT) should be examined. Preterm infants often have low PMT, but seem to develop exaggerated AMP. This behaviour is most obvious in the trunk and legs and has often been described as hyperextension in preterm infants after term age [30–32].

Despite the poorer muscle tone in preterm infants, the present study did not identify significantly greater postural asymmetries in preterm compared to full-term children.

In infants born preterm, immaturity of the system may lead to an asymmetrical performance and posture even when these infants are healthy [33–36]. A strong preference to turn the head to the right side and subtle asymmetries in fetal movements in infants born preterm have been described previously [25, 29, 37–40]. The high prevalence of a positional preference in infants born preterm at term equivalent age requires extra alertness to prevent the development of a deformational plagiocephaly, especially in boys and twins [41]. Yet, as it was found in the present study, at a later developmental age differences in frequency of asymmetries tend to decrease, regardless of sex, which may be linked with reduced asymmetries resulting from early interventions administered to preterm infants if asymmetry is identified after birth [41].

Small asymmetries, up to 1 cm, in the trunk region, identified by noninvasive assessments are considered to be rather common [42, 43]; therefore just like symmetry, slight asymmetry may be recognized as typical and normal in the structure of human body. In the present study the mean size of asymmetries did not exceed 8 mm. Yet, a continued tendency to tilt the body towards one side, in challenging conditions, such as pubertal growth spurt, may lead to increased asymmetry [42]. Premature birth does not predispose to greater asymmetries or their orientation; therefore these authors believe the factor can be disregarded in qualification for comparative screening studies. The identified tendency in asymmetry orientation makes it possible to prevent pathologic asymmetry.

The presented study is the first detailed assessment of body posture in preterm children reported in the literature. Moreover, the study was carried out among subjects at six years of age, with measurements in standing and sitting position and with analysis of asymmetry orientation. Of significance here is also the inclusion criterion qualifying children born at gestational age of up to 32 weeks.

### 4.1. Limitations

A limitation of the study relates to the lack of analysis relative to the degree of prematurity at birth and size of asymmetry, yet this was not taken into account due to the fact that the results do not show statistical significance. Given the scarcity of the related evidence, similar research, however, should be continued taking into account the relationship between asymmetry and other factors, such as age, level of motor activity, sex, and degree of pathological asymmetry. Additionally it would be worthwhile to compare results between standing and sitting position, because examination of the surface morphology of the trunk in the sitting position is performed when a leg length inequality is present, in order to reveal true asymmetry and not the one which comes from tilting of the body in the standing position. Such analyses have not been conducted in the present study as no leg length asymmetries were identified among the participants.

Assessment designed in this way would identify not only relationship of asymmetry to age or motor activity, but also the correlation between the size of asymmetry and the relevant factors.

## 5. Conclusions

1. There are no significant differences in body posture in the coronal plane, between preterm children and full-term children.
2. Premature birth does not have adverse effects related to body posture asymmetry in preterm children at the age of six.

## Figures and Tables

**Table 1:** Anthropometric parameters in the cohort.

Variables	Preterm group (*n* = 50)			Control group (*n* = 51)			*Z*	*p*
	$\overset{-}{x}$	Me	SD	$\overset{-}{x}$	Me	SD		
Body height	116.52	115.50	8.02	118.98	118.00	7.91	−1.82	0.069
Body weight	21.11	19.90	4.78	23.12	21.90	4.74	−**2.57**	**0.010**
BMI	15.40	15.11	2.05	16.20	15.80	1.94	−**2.35**	**0.019**

*n* : number of observations; $\overset{-}{x}$: arithmetic mean; Me: median; SD: standard deviation.

**Table 2:** Body posture parameters in standing and in sitting position, relative to gestational age at birth.

Variables	Preterm group (*n* = 50)			Control group (*n* = 51)			*t*/*Z*	*p*
	$\overset{-}{x}$	Me	SD	$\overset{-}{x}$	Me	SD		
Standing position								
CIT [degree]	1.26	0.95	0.91	1.18	1.10	0.82	0.16	0.876
DHS [mm]	7.17	5.60	5.40	5.66	5.00	4.60	1.30	0.194
DHCS [mm]	5.40	5.05	3.71	5.57	4.50	4.88	0.36	0.721
DDCS [mm]	5.18	4.50	4.36	5.10	3.90	4.65	0.36	0.721
DHP [mm]	2.50	2.20	2.02	2.84	2.80	2.26	−0.71	0.480
AS [mm]	5.43	4.95	2.63	5.38	4.50	3.23	0.61	0.541

Sitting position								
CIT [degree]	1.64	1.60	1.12	1.59	1.50	1.17	0.34	0.734
DHS [mm]	5.54	4.50	5.43	6.51	5.00	5.75	−0.91	0.365
DHCS [mm]	5.27	4.50	3.89	4.93	4.50	4.29	0.67	0.505
DDCS [mm]	6.34	5.10	5.05	7.88	6.80	5.71	−1.32	0.185
DHP [mm]	2.79	2.20	2.44	3.79	3.30	3.31	−1.37	0.172
AS [mm]	6.94	6.50	3.03	7.02	6.30	4.18	0.49	0.621

*n*: number of observations; $\overset{-}{x}$: arithmetic mean; Me: median; SD: standard deviation; *t*: result of Student's *t*-test for independent variables; *Z*: result of Mann–Whitney *U* test; *p*: level of probability; CIT [degree]: coronal inclination of the trunk; DHS [mm]: difference in the heights of shoulders; DHCS [mm]: difference in the heights of the lower corners of the scapulae; DDCS [mm]: difference in the distances from the lower corners of the scapulae to the spine; DHP [mm]: difference in the height of the pelvis; SA [mm]: maximum deflection of the line connecting the spinous processes from C7-S1 line.

**Table 3:** Body posture parameters in standing and in sitting position, relative to gestational age at birth, in girls.

Variables	Girls preterm group (*n* = 25)			Girls control group (*n* = 29)			*t*/*Z*	*p*
	$\overset{-}{x}$	Me	SD	$\overset{-}{x}$	Me	SD		
Standing position								
CIT [degree]	1.27	1.10	0.86	0.99	1.00	0.77	1.20	0.230
DHS [mm]	6.16	4.50	5.58	4.67	2.80	4.18	0.97	0.330
DHCS [mm]	5.55	5.60	3.77	5.66	4.50	4.35	0.20	0.841
DDCS [mm]	5.93	5.10	4.64	4.13	2.80	4.14	1.88	0.060
DHP [mm]	2.37	1.70	2.09	3.02	2.80	2.25	−1.01	0.311
AS [mm]	4.82	4.50	2.29	5.47	4.90	3.43	−0.39	0.696

Sitting position								
CIT [degree]	1.65	1.60	1.13	1.43	1.20	1.16	0.68	0.500
DHS [mm]	4.62	3.90	4.47	4.80	3.30	4.61	−0.20	0.841
DHCS [mm]	5.77	5.00	4.22	4.52	4.50	3.98	1.07	0.285
DDCS [mm]	5.79	4.65	4.41	6.59	6.80	4.54	0.63	0.531
DHP [mm]	2.71	2.20	2.26	3.79	3.30	3.10	−1.12	0.261
AS [mm]	6.46	6.10	3.02	6.60	5.60	3.43	−0.15	0.882

*n*: number of observations; $\overset{-}{x}$: arithmetic mean; Me: median; SD: standard deviation; *t*: result of Student's *t*-test for independent variables; *Z*: result of Mann–Whitney *U* test; *p*: level of probability; CIT [degree]: coronal inclination of the trunk; DHS [mm]: difference in the heights of shoulders; DHCS [mm]: difference in the heights of the lower corners of the scapulae; DDCS [mm]: difference in the distances from the lower corners of the scapulae to the spine; DHP [mm]: difference in the height of the pelvis; SA [mm]: maximum deflection of the line connecting the spinous processes from C7-S1 line.

**Table 4:** Body posture parameters in standing and in sitting position, relative to gestational age at birth, in boys.

Variables	Boys preterm group (*n* = 25)			Boys control group (*n* = 22)			*t*/*Z*	*p*
	$\overset{-}{x}$	Me	SD	$\overset{-}{x}$	Me	SD		
Standing position								
CIT [degree]	1.24	0.90	0.97	1.44	1.25	0.84	−1.05	0.295
DHS [mm]	8.18	8.90	5.12	6.97	5.60	4.90	0.90	0.370
DHCS [mm]	5.24	4.50	3.71	5.46	3.30	5.60	0.42	0.677
DDCS [mm]	4.43	3.40	4.01	6.39	6.90	5.05	−1.30	0.193
DHP [mm]	2.64	2.20	1.98	2.59	1.95	2.31	0.31	0.756
AS [mm]	6.04	5.10	2.85	5.27	4.20	3.01	1.08	0.281

Sitting position								
CIT [degree]	1.62	1.45	1.14	1.80	1.60	1.18	−0.37	0.715
DHS [mm]	6.54	5.05	6.29	8.76	7.25	6.39	−1.25	0.211
DHCS [mm]	4.71	4.50	3.52	5.47	5.05	4.70	−0.27	0.791
DDCS [mm]	6.94	6.10	5.74	9.60	8.35	6.68	−1.62	0.104
DHP [mm]	2.88	1.95	2.69	3.80	2.75	3.64	−0.76	0.446
AS [mm]	7.46	7.60	3.04	7.58	6.35	5.03	0.63	0.529

*n*: number of observations; $\overset{-}{x}$: arithmetic mean; Me: median; SD: standard deviation; *t*: result of Student's *t*-test for independent variables; *Z*: result of Mann–Whitney *U* test *p*: level of probability; CIT [degree]: coronal inclination of the trunk; DHS [mm]: difference in the heights of shoulders; DHCS [mm]: difference in the heights of the lower corners of the scapulae; DDCS [mm]: difference in the distances from the lower corners of the scapulae to the spine; DHP [mm]: difference in the height of the pelvis; SA [mm]: maximum deflection of the line connecting the spinous processes from C7-S1 line.

**Table 5:** Coronal plane inclinations in the trunk region, relative to gestational age at birth and position of the body during measurement.

Variables	Preterm group (*n* = 50)		Control group (*n* = 51)		*χ* ^2^/*p*	Preterm group (*n* = 42)		Control group (*n* = 51)		*χ* ^2^/*p*
	*n*	%	*n*	%		*n*	%	*n*	%	
	Standing position					Sitting position				
CIT										
Left	26	52.0	25	49.0	*χ* ^2^ = 1.19 *p* = 0.550	10	23.8	20	39.2	*χ* ^2^ = 2.50 *p* = 0.286
Right	23	46.0	26	51.0		29	69.1	28	54.9	
Centre	1	2.0	0	0.0		3	7.1	3	5.9	
DHS										
Left lower than right	22	44.0	22	43.2	*χ* ^2^ = 0.00 *p* = 0.995	8	19.1	14	27.5	*χ* ^2^ = 1.50 *p* = 0.471
Right lower then left	26	52.0	27	52.9		26	61.9	31	60.8	
Centre	2	4.0	2	3.9		8	19.1	6	11.8	
DHCS										
Left lower than right	19	38.0	17	33.3	*χ* ^2^ = 0.25 *p* = 0.880	15	35.7	16	31.4	*χ* ^2^ = 0.34 *p* = 0.839
Right lower then left	28	56.0	31	60.8		23	54.8	31	60.8	
Centre	3	6.0	3	5.9		4	9.5	4	7.8	
DDCS										
Left scapula closer to spine than the right one	28	56.0	25	49.0	*χ* ^2^ = 0.52 *p* = 0.769	27	64.3	29	56.9	*χ* = 0.59 *p* = 0.741
Right scapula closer to spine than the left one	20	40.0	24	47.1		14	33.3	20	39.2	
The same distance	2	4.0	2	3.9		1	2.4	2	3.9	
DHP										
Left iliac spine lower than the right one	16	32.0	12	23.5	*χ* ^2^ = 2.22 *p* = 0.328	16	38.1	20	39.2	*χ* ^2^ = 1.73 *p* = 0.419
Right iliac spine lower than the left one	29	58.0	29	56.9		18	42.9	26	51.0	
The same height	5	10.0	10	19.6		8	19.1	5	9.8	
SA										
Left	35	70.0	34	66.7	*χ* ^2^ = 0.12 *p* = 0.718	29	69.1	40	78.4	*χ* ^2^ = 1.05 *p* = 0.303
Right	15	30.0	17	33.3		13	31.0	11	21.6	

*n*: number; %: per cent *χ*^2^: result of Pearson's chi-squared test; *p*: level of probability; CIT [degree]: coronal inclination of the trunk; DHS [mm]: difference in the heights of shoulders; DHCS [mm]: difference in the heights of the lower corners of the scapulae; DDCS [mm]: difference in the distances from the lower corners of the scapulae to the spine; DHP [mm]: difference in the height of the pelvis; SA [mm]: maximum deflection of the line connecting the spinous processes from C7-S1 line.
